# Canonical Bone Morphogenetic Protein Signaling Regulates Expression of Aquaporin-4 and Its Anchoring Complex in Mouse Astrocytes

**DOI:** 10.3389/fncel.2022.878154

**Published:** 2022-04-20

**Authors:** Nadia Skauli, Ekaterina Savchenko, Ole Petter Ottersen, Laurent Roybon, Mahmood Amiry-Moghaddam

**Affiliations:** ^1^Division of Anatomy, Department of Molecular Medicine, Institute of Basic Medical Sciences, University of Oslo, Oslo, Norway; ^2^Stem Cell Laboratory for CNS Disease Modeling, Department of Experimental Medical Science, BMC D10, Lund University, Lund, Sweden; ^3^Karolinska Institutet, Stockholm, Sweden

**Keywords:** astrocyte, aquaporin-4, bone morphogenetic protein, Smad1/5/9, dystrophin

## Abstract

Aquaporin-4 (AQP4) is the predominant water channel in the brain; it is enriched in astrocytic foot processes abutting vessels where it is anchored through an interaction with the dystrophin-associated protein (DAP) complex. Enhanced expression with concomitant mislocalization of AQP4 along astrocyte plasma membranes is a hallmark of several neurological conditions. Thus, there is an urgent need to identify which signaling pathways dictate AQP4 microdistribution. Here we show that canonical bone morphogenetic proteins (BMPs), particularly BMP2 and 4, upregulate AQP4 expression in astrocytes and dysregulate the associated DAP complex by differentially affecting its individual members. We further demonstrate the presence of BMP receptors and Smad1/5/9 pathway activation in BMP treated astrocytes. Our analysis of adult mouse brain reveals BMP2 and 4 in neurons and in a subclass of endothelial cells and activated Smad1/5/9 in astrocytes. We conclude that the canonical BMP-signaling pathway might be responsible for regulating the expression of AQP4 and of DAP complex proteins that govern the subcellular compartmentation of this aquaporin.

## Introduction

AQP4 is one of the most prevalent membrane proteins in astrocytes; it is concentrated in endfoot membranes abutting brain microvessels (Amiry-Moghaddam and Ottersen, 2003; Papadopoulos and Verkman, 2013). AQP4 is anchored to these membrane domains through an interaction with α-syntrophin, a member of the dystrophin associated protein complex (DAPC) (Neely et al., 2001).

Studies of mice that suffer a loss of perivascular AQP4 following genetic deletion of α-syntrophin have shown that this pool of AQP4 is involved in a number of critical functions including potassium homeostasis (Amiry-Moghaddam et al., 2003b) and edema formation following stroke (Amiry-Moghaddam et al., 2003a), or hyponatremia (Amiry-Moghaddam et al., 2004b). In addition, studies of AQP4 knockout mice have shown that AQP4 may play a pro-inflammatory role in different mouse models of neurological diseases (Li et al., 2011; Prydz et al., 2020).

Several neurological conditions are associated with a dysregulation of AQP4 expression. In an early study, we found AQP4 to be strongly reduced in perivascular astroglial endfeet following transient cerebral ischemia in mice (Amiry-Moghaddam et al., 2003a). A detailed analysis using immunogold cytochemistry revealed a complex pattern of changes after transient middle cerebral artery occlusion: the striatal core displayed a permanent loss of perivascular AQP4 while the most affected part of the cortex revealed a loss of perivascular AQP4 followed by a partial recovery (Frydenlund et al., 2006). Changes of this nature are likely to impact the build-up and resolution of brain edema after an ischemic insult (Papadopoulos et al., 2004; Nagelhus and Ottersen, 2013).

A loss of perivascular AQP4 similar to that found in experimental stroke—albeit somewhat less pronounced, was observed in the brain of patients that had suffered from mesial temporal lobe epilepsy (Eid et al., 2005). Interestingly, the same brains showed an increase in the total level of AQP4 (ibid., also see Lee et al., 2004) pointing to a dysregulation of AQP4 expression. In an experimental study of kainate induced epilepsy in rats, loss of perivascular AQP4 preceded the development of chronic seizures, suggesting that this loss might be of pathophysiological relevance for epileptogenesis, and not just an effect of the epileptic condition (Alvestad et al., 2013).

Dysregulation of AQP4 expression is also a distinct feature of Alzheimer’s disease (AD) and experimental models of this disease (Pérez et al., 2007; Wilcock et al., 2009; Yang et al., 2011; Valenza et al., 2019). In the ArcSwe model of AD, there is a conspicuous loss of AQP4 from endfeet associated with perivascular amyloid deposits, while endfeet contacting normal looking vessels appeared to retain their pool of AQP4 (Yang et al., 2011). This latter study (Yang et al., 2011) showed that the loss of perivascular AQP4 was accompanied by an increase in the total level of AQP4, mimicking the situation in human mesial temporal lobe epilepsy (Eid et al., 2005). The pattern that emerges is that the depletion of perivascular AQP4 seen in a variety of neurological conditions is not secondary to a general downregulation of AQP4 expression. On the contrary, loss of perivascular AQP4 is typically coupled to an increased level of AQP4 in the tissue. This reflects a mislocalization of AQP4, i.e., a redistribution of AQP4 from endfeet membranes to other astroglial membrane domains. Such a mislocalization can be reproduced experimentally by targeted deletion of α-syntrophin or other members of the dystrophin complex that serves to anchor AQP4 in endfoot membranes (Yokota et al., 2000; Neely et al., 2001; Vajda et al., 2002; Amiry-Moghaddam et al., 2004a; Valenza et al., 2019).

The realization that dysregulation of AQP4 expression figures prominently in numerous neurological conditions, emphasizes the need to uncover the mechanisms governing the expression level and subcellular localization of this water channel. There is a dearth of knowledge on this issue. Evidence has accrued to suggest that soluble factors control AQP4 expression (Camassa et al., 2015) and that p38 might be involved (Nito et al., 2012; Li et al., 2019).

Here we explore the hypothesis that bone morphogenetic proteins (BMPs) regulate the expression and subcellular distribution of AQP4. BMPs are a highly conserved and diverse family of growth factors mainly studied for their roles in development. A recent study has shown that overexpression of BMP7 during development upregulates the expression of AQP4 in the brain through Smad1 phosphorylation (Morita et al., 2021). Other studies have uncovered novel roles for BMPs in the adult CNS coupled to a number of conditions and processes including aging and repair (Hart and Karimi-Abdolrezaee, 2020). Interestingly, BMP4, a canonical BMP activating the Smad1/5/9 signaling pathway, is increased by several fold in aging mouse hippocampus (Meyers et al., 2016). Moreover, raised levels of BMP2 and BMP4 have been implicated in pathological conditions such as stroke (Shin et al., 2012) and AD (Meyers et al., 2016). In the present study we show that astrocytes possess BMP receptors, and that BMPs lead to upregulation of AQP4 expression and dysregulation of members of the dystrophin associated protein complex (DAPC) responsible for the anchoring of AQP4 to perivascular membrane domains. We conclude that BMP signaling regulates AQP4 expression and microdistribution and—by inference—that perturbed BMP signaling could contribute to the mislocalization of AQP4 that has emerged as a common denominator of a range of neurological disorders.

## Materials and Methods

### Animals

Adult, newborn and embryonic mice of the C57/BL6J strain (Jackson Laboratories, Boulder, CO, United States) were used in this study. The mice were allowed *ad libitum* access to food and drinking water. Experimental protocols were approved by the Norwegian Animal Research Authority (NARA) with license number FOTS 8572 and carried out in accordance with the European Directive 2010/63/EU. The derivation of mouse embryonic stem cells (mESC) was approved by the ethical committee for the use of laboratory animals at Lund University and the Swedish Work Environment Authority with permit number M43-15 and carried out in accordance with European and Swedish regulations.

### Derivation of Mouse Embryonic Stem Cell and Maintenance

Wildtype (C57BL/6) mouse embryonic stem cell (mESC) line was derived as previously described (Chumarina et al., 2017). mESCs (lines CSC 2.3), at passages 14 and 17, were cultured on a monolayer of CF1 irradiated MEFs in DMEM medium containing 15% fetal bovine serum (FBS), penicillin/streptomycin (P/S; 100 Units/mL and 100 mg/mL, respectively), 2 mM L-glutamine (GIBCO), 100 mM non-essential amino acids (NEAA, GIBCO), 1% nucleosides (Millipore), 10% FBS (Millipore), β-mercaptoethanol (110 μM), and supplemented with leukemia inhibitor factor (LIF, 10,000 units/mL; Millipore, ESG1106). Cells were maintained at 37 °C, 5% CO_2_, and passaged using 0.5% trypsin (Millipore) every 4–5 days.

### Generation of Mouse Embryonic Stem Cell-Derived Astrocytes

mESC-derived astrocytes were generated as previously described (Roybon et al., 2013; Savchenko et al., 2019). Briefly, mESCs were harvested by trypsinization, transferred to low-attachment culture flasks and cultured as free-floating spheres in 50% of DMEM and 50% of Neurobasal media containing penicillin/streptomycin (P/S; 100 Units/mL and 100 mg/mL, respectively), 2 mM L-glutamine (GIBCO), β-mercaptoethanol (110 μM) and 10% KnockOut Serum Replacement (Invitrogen). The medium was supplemented with 1 μM Retinoic acid (RA) (Sigma-Aldrich, R2525) and 0.5 μM Smoothened Agonist (SAG) (Millipore, 566660) and changed at days 2 and 5. On day 7, the free-floating spheres were mechanically dissociated and transferred to neurosphere medium composed of DMEM/F12 (Thermo Fisher Scientific), 2% B27 supplement, 2 mM L-glutamine, 100 mM non-essential amino acids, P/S (100 Units/mL and 100 mg/mL, respectively) and 2 mg/mL heparin (Sigma-Aldrich), supplemented with FGF2 (20 ng/mL) and EGF (20 ng/mL) for 2 weeks. Additionally, spheres were passaged on day 14 to generate secondary neurospheres. On day 21, spheroids were dissociated into single cells and plated onto polyornithine (100 μg/mL)/laminin (2 μg/mL) coated plates in neurosphere medium supplemented with 10% Fetal Bovine Serum (FBS) (Thermo Fisher Scientific, 10270106). The medium was changed twice per week. On day 36, the cells were used for the experiments.

### Generation of Primary Murine Astrocyte Cultures

Murine astrocyte cultures were prepared from C57/BL6J embryonic E16-18 or P1-P2 mouse cortices by a protocol modified from de Vellis and Cole (2012); Pacifici and Peruzzi (2012), and Schildge et al. (2013). The skin was removed, and skull opened with separate sterile scissors to expose the brain, and the meninges removed under a stereomicroscope with sterile filter paper. Approximately 1 mm x 1 mm pieces of cortex were extracted with a sterile scalpel from each brain hemisphere and collected in cold complete medium containing 10% FBS (BioWest, Nuaillé, France), 90% DMEM High glucose (BioWest) and 1% Penstrep (Thermo Fisher Scientific, Waltham, MA, United States). Cortex pieces were washed with cold complete medium two times before enzymatic digestion with warm 1–2 mL TrypLE Express (Thermo Fisher Scientific) for 10 min in a 37^°^C water bath with occasional shaking. Complete medium was added, and the tubes were centrifuged for 5 min at 1,000 rpm. The pellet was triturated in complete medium using first a 10 mL then 5 mL stripette. The cell suspension was filtered through a 70 μm cell strainer to remove debris. The filtered cells were plated at a density of 3–5 × 10^6^ cells per flask. Care was taken to complete the process within 1–2 h. Medium was changed on day 2 after plating, and every 3 days thereafter. After 7–8 days, astrocytes were confluent and were shaken in warm serum free medium for several minutes to remove contaminating microglia and oligodendrocytes, which grow on top of the astrocyte monolayer. The serum free media containing contaminating cells was removed, the astrocyte monolayer washed twice, trypsinized, and centrifuged at 180 g for 5 min. The cells were resuspended at a 1:2 ratio into T75 flasks and cultured in medium with serum. To ensure matured astrocytes, experiments were performed 2–3 weeks after the first split, and passages were limited to three (Schildge et al., 2013). For all experiments, astrocytes were grown on high molecular weight (Mol W > 300,000) poly-D-lysine (Merck Millipore, Darmstadt, Germany) coated flasks and plates and cultured in a 5% CO_2_ 37^°^C 95% O_2_ humidity incubator. For BMP treatments, primary astrocytes were grown to 90% confluence on 96-well glass plates (for Plate Runner assays), 24-well plates (for RNA extraction) or 6-well plates (for protein extraction). The cells were washed with PBS and kept in serum free media overnight. The next day, cells were treated with 50 ng/mL BMPs in serum free media for 6 days, 72 or 12 h.

### Trophos^HD^ Plate Runner Imaging

We assessed BMPs –2, –3, –4, –5, –6, –7, and –10 for their effect on AQP4 and GFAP protein expression in mouse embryonic cell derived astrocytes (mESC) and primary astrocytes through a high throughput immunofluorescent assay (Plate Runner HD, Trophos), as previously described (Savchenko et al., 2019). Astrocytes were seeded in 96-well plates in serum free medium and treated with 50 ng/μL recombinant human BMP1 (1927-ZN, R&D Systems, MN, United States), BMP2 (120-02C, Peprotech), BMP3 (120-24B, Peprotech), BMP4 (PHC9531, Thermo Fisher Scientific), BMP5 (120-39, Peprotech), BMP6 (120-06, Peprotech), BMP7 (120-03P, Peprotech), BMP10 (120-40 Peprotech), FGF2 as a negative control (100-18B, Peprotech) or in BMP-free medium (condition used as a negative control for 6 days, before fixation in 4% PFA in PBS followed by a 1 h blocking with 5% BSA in PBS. The fixed cells were immunolabeled overnight (Table 1), washed with PBS and incubated with secondary antibodies 1:500 anti-chicken Alexa Fluor 488 (Thermo Fisher Scientific Cat# A-11039, RRID:AB_2534096) and anti-rabbit Alexa Fluor 555 (Thermo Fisher Scientific Cat# A27039, RRID:AB_2536100) for 1 h at room temperature. DAPI (Thermo Fisher Scientific) diluted 1:50,000 in 1× PBS was used to stain nuclei. Duplicate wells from three separate experiments with astrocyte culture originating from two separate batches of embryonic cortices isolated on the same day were used. After imaging on Plate Runner HD (Trophos), images were analyzed by the Metamorph (Cell Scoring Application Module; Molecular Devices) analysis software. Data is presented as mean average cell intensities of staining.

**TABLE 1:** Antibodies used for immunostaining.

Protein	Origin	Category ID	Vendor	WB	IF	RRID
AQP4	Rabbit	A5971	Sigma-Aldrich	1:5,000	1:500	RRID:AB_258270
BMP2	Rabbit	ab14933	Abcam	1:1,000	1:100	RRID:AB_2243574
BMP4	Rabbit	ab39973	Abcam	1:1,000	1:100	RRID:AB_2063523
GAPDH	Mouse	ab9484	Abcam	1:1,000	NA	RRID:AB_307274
GFAP	Chicken	PCK-591P	Nordic BioSite	NA	1:500	RRID:AB_291542
GFAP	Mouse	MAB360	Chemicon	1:1,000	NA	RRID:AB_11212597
MAP2	Chicken	ab5392	Abcam	NA	1:500	RRID:AB_2138153
NeuN	Mouse	MAB377	Millipore	NA	1:500	RRID:AB_2298772
pSmad1/5/9	Rabbit	13820S	Cell Signaling	1:1,000	NA	RRID:AB_2493181
Smad1	Rabbit	6944S	Cell Signaling	1:1,000	NA	RRID:AB_10858882
α-tubulin	Rabbit	ab4074	Abcam	1:2,000	NA	RRID:AB_2288001
DAT	Rat	sc-32258	Santa Cruz	NA	1:500	RRID:AB_627400

### Regional Dissection of Adult Mouse Brains

Adult (4-month-old) male C57/BL6J mice (*n* = 4) were anesthetized with isofluorane (Baxter, Deerfield, IL, United States) and decapitated before regional dissection of frontal cortex, striatum, parietal cortex, hippocampus, midbrain and cerebellum was performed on ice and the regions flash frozen in liquid nitrogen. Regions from each hemisphere were stored at –80C and used for RNA or protein extraction, respectively.

### RNA Extraction, cDNA Synthesis and RT-qPCR

Gene expression measurement was performed as previously described (Rao et al., 2019) with some modifications. For primary astrocyte samples, RLT lysis buffer was added directly to plate and scraped. Total RNA was extracted using the RNeasy^®^ Plus Mini Kit (QIAGEN) and RNA concentrations were quantified by NanoDrop (Thermo Fisher Scientific). 50 ng RNA was reverse transcribed into cDNA by Oligo (dT)15 primers using the GoScript Reverse Transcription System (Promega) in a reaction volume of 20 μL. For adult mouse brain regions, regions were homogenized in RLT buffer and total RNA extracted using the RNeasy^®^ Plus Mini Kit and RNA concentrations were quantified by NanoDrop. 500 ng total RNA was reverse transcribed to cDNA as described above. The cDNA was diluted in 10 mM Tris-HCl (pH 8.0). Real time PCR was performed usingSYBR^®^ Green PCR Master Mix (Applied Biosystems) with specific primers at 200 nM concentration. Real time assays were carried out in 96 well plates using the StepOnePlus™ Real Time PCR System (v2.3, Applied Biosystems). Thermal cycling was performed on the StepOnePlus system (Applied Biosystems) under the following conditions: 95°C for 10 min, followed by 40 cycles of 95°C for 15 s and 60°C for 60 s. 1.25–5 ng input was used for RT-qPCR depending on gene expression. Copy numbers were calculated according to an absolute standard for quantification. Copy numbers were determined by calculating copy numbers per ng RNA input into the cDNA synthesis, using the formula: Copy number/ng = [A]/Mw, where [A] is the Avogadro’s number (6.02 × 10^23^/mol) and Mw is the molecular weight of the amplicon (in ng/mol). Oligocalc (Kibbe, 2007) was used to calculate the molecular weight of the amplicon. Thereafter, a standard curve was prepared for each primer with 10-fold serial dilutions. NormFinder (Andersen et al., 2004) was used to identify the best housekeeping gene or combination of housekeeping genes. For primary astrocyte samples *Tbp, Gapdh, Actb*, *Hprt1*, *Ubc*, *Ppia*, and *H2afz* were analyzed, and the best stability value of 0.063 was achieved with a combination of *Ppia* and *H2afz*. These genes were used for double normalization of the data. For adult mouse brain regions Housekeeping genes *Tbp, Ppia, H2afz, Hprt1*, and *Actb* were evaluated by NormFinder. *Ppia* alone was found to have the best stability value of 0.069 and was used for normalization. Non-reverse transcriptase (NRT) and non-template (NTC) controls were included on each plate. One-way ANOVA *post hoc* LSD test was used for statistical analysis. Data are presented as mean values ± SEM. *P* < 0.05 was considered as significant. Primers used are listed in Table 2.

**TABLE 2:** Primers used in RT-qPCR analysis.

Gene	Forward primer	Reverse primer	Size (bp)
*Actb*	5′-GCTCTTTTCCAGCCTTCCTT-3′	5′-GTGCTAGGAGCCAGAGCAGT-3′	194
*Acvr1*	5′-TGTTGGAGTGTGTCGGGAAG-3′	5′-CACAGCTGGGTACTGGAGTG-3′	211
*Acvr1b*	5′-GGTGGGGACCAAACGATACA-3′	5′-TCGGAGGGCACTAAGTCGTA-3′	189
*Acvr1l*	5′-ACATCCTAGGCTTCATCGCC-3′	5′-ATGGCTGGTTTGCCTTGAGT-3′	214
*Acvr2a*	5′-TGGCCAGCATCCATCTCTTG-3′	5′-GCATTGCCATTCCTGCATGT-3′	104
*Acvr2b*	5′-CCCACTTCAGGACAAGCAGT-3′	5′-CAGGTTGGAGCCTCGTTTCT-3′	115
*Aqp4*	5′-TTTGGACCCGCAGTTATCAT-3′	5′-GTTGTCCTCCACCTCCATGT-3′	203
*Bmp10*	5′-GAACGAAGATCTGTTTTCTCAACCA-3′	5′-ATGCGATCTCTCTGCACCAG-3′	141
*Bmp2*	5′-CACCCCAAGACACAGTTCCC-3′	5′-AACACTAGAAGACAGCGGGT-3′	216
*Bmp4*	5′-CGAGCCAACACTGTGAGGAGTTTC-3′	5′-TTCTCTGGGATGCTGCTGAGGTT-3′	113
*Bmp5*	5′-AGCACCAGAAGGGTATGCTG-3′	5′-ACATCAGGTGTACCAGGGTCT-3′	113
*Bmp6*	5′-TGTGAACCTGGTGGAGTACG-3′	5′-AAACTCCCCACCACACAGTC-3′	147
*Bmp7*	5′-GGCCTGCAAGAAACATGAGC-3′	5′-AGTGAACCAGTGTCTGGACG-3′	170
*Bmpr1a*	5′-TGACCTGGGCCTAGCTGTTA-3′	5′-ATTCCTCCACGATTCCTCCTG-3′	221
*Bmpr1b*	5′-CCAAGCGCTATATGCCTCCA-3′	5′-TGGGGAATGAAGGCCGTAAC-3′	243
*Bmpr2*	5′-AAGCTGCTGGAGCTGATTGG-3′	5′-AACTGGACGCTCATCCAAGG-3′	72
*Dag1*	5′-CGGATGAGCTGGATGACTCT-3′	5′-TGATAGGGAGGTGCGTTAGG-3′	190
*Dmd (DP71)*	5′-CAAGCTTACTCCTCCGCTCT-3′	5′-GAGCCTTCTGAGCTTCATGG-3′	196
*Dtna*	5′-TTGGAACGTCATTGAAGCATTGC-3′	5′-GCATCCTCTTGTTGAGCTGGT-3′	125
*Gfap*	5′-GCACTCAATACGAGGCAGTG-3′	5′-GCTCTAGGGACTCGTTCGTG-3′	207
*H2afz*	5′-ACAGCGCAGCCATCCTGGAGTA-3′	5′-TTCCCGATCAGCGATTTGTGGA-3′	202
*Hprt1*	5′-GCCCCAAAATGGTTAAGGTT-3′	5′-TTGCGCTCATCTTAGGCTTT-3′	208
*Ppia*	5′-CGCGTCTCCTTCGAGCTGTTTG-3′	5′-TGTAAAGTCACCACCCTGGCACAT-3′	150
*Snta1*	5′-GCTGGCTGACAGAACAGTTG-3′	5′-TTCTGCATCATAGGGCACTG-3′	197
*Ubc*	5′-CGTCGAGCCCAGTGTTACCACCAAGAAGG-3′	5′-CCCCCATCACACCCAAGAACAAGCACAAG-3′	112

### Protein Extraction, Sodium Dodecyl Sulfate-Polyacrylamide Gel Electrophoresis and Western Blotting

Experiments were performed as previously described with some modifications (Katoozi et al., 2020). Total protein extraction was performed by homogenization in RIPA buffer with freshly added SigmaFAST protease inhibitor (Sigma-Aldrich, St. Louis, MO, United States) and PhosSTOP phosphatase inhibitor (Roche Life Science, Basel, Switzerland). For primary astrocyte cultures, lysis buffer was added directly in the wells and the cells collected by scraping. For adult mouse brain regions, samples were homogenized in Eppendorf tubes. The homogenates were incubated on ice for 30 min with occasional vortexing before centrifugation at 22,000 g at 4°C for 15 min. The supernatant was collected, and protein concentrations measured using a Pierce™ BCA protein assay kit (Thermo Fisher Scientific, Waltham, MA, United States). Briefly, samples containing 10 μg protein (primary astrocytes) or 40 μg protein (brain samples) were heated in 1 × Laemmli sample buffer at 37°C for 15 min and separated on Criterion™ 18- or 26-well gels (BioRad, Hercules, CA, United States) by sodium dodecyl sulfate-polyacrylamide gel electrophoresis (SDS-PAGE) using the Criterion™ (BioRad) Tris-glycine system at 160 V for 1 h 15 min. Proteins were transferred to 0.2 μm Immun-Blot PVDF membranes by wet blotting at 100 V for 45 min at 4°C (BioRad). Uniform transfer of proteins was verified by reversible Ponceau S staining (0.1%w/v, 1% acetic acid, Sigma-Aldrich). Membranes were blocked for 1 h at RT in 3% BSA in 1 × Tris-buffered saline (TBS) (BioRad), washed in 1 × TBS with 1% Tween-20 (Sigma-Aldrich) and cut before separate overnight incubation at 4°C with primary antibodies (Table 1). Subsequently, membranes were incubated with anti-igG Horseradish Peroxidase conjugated antibodies; rabbit (GE Healthcare Cat# NA934, RRID:AB_772206) or mouse (GE Healthcare Cat# NA931, RRID:AB_772210) secondary antibodies at 1: 20,000 dilution for 1 h at room temperature, washed in TBST 3 × 10 min and immunoreactive bands detected by SuperSignal™ West Pico Chemiluminescent Substrate (Thermo Fisher Scientific) on a ChemiDoc™ Touch (BioRad) imaging system and bands quantified as arbitrary background-subtracted density units in Image Studio Lite (Ver 5.2, Licor Biosciences, Nebraska, United States). For blots with unspecific background labeling, background correction was performed by automated computation of the median intensity of pixels in an area adjacent to individual bands and subtracting it from the band intensities. Normalization was performed by dividing intensities of protein bands of interest with the normalizing control band intensity for their respective laneRabbit anti-α-tubulin (Abcam; Cat# ab4074, RRID:AB_2288001) and GAPDH (Abcam Cat# ab9484, RRID:AB_307274) were used as normalizing controls for primary astrocyte and brain regions, respectively. The obtained values were transferred to SPSS Version 25 (SPSS, Chicago, IL, United States) and compared using ANOVA *post hoc* LSD. Data are presented as mean values ± SEM. *P* < 0.05 was considered as significant.

### Immunofluorescence

For light microscopic immunofluorescence experiments of BMP2 and BMP4 expression, adult (3–4 months) C57/BL6J male (*n* = 4) and female (*n* = 1) mouse brains were harvested and fresh frozen in cooled isopentane on dry ice before sectioning by cryostat to 20 or 40 μm sections on glass slides. Fresh frozen sections were thawed for 5 min and fixation was carried out using 4% formaldehyde in 0.1 M PB at pH 6.0. For investigations of pSmad1/5/9 expression, animals (*n* = 4) were anesthetized with zoletil mixture (0.2 mL/10 g; i.p.) and transcardially perfused fixed with 2% ice cold dextran, followed by 15 min of 4% formaldehyde (FA), freshly depolymerized from paraformaldehyde (PFA), dissolved in 0.1 M phosphate buffer (PB). Perfused brains were post fixed for 24 h and treated with a 10–30% sucrose gradient before cryostat sectioning. Sections from perfused animals were thawed for 15 min before use. Sections were rinsed with phosphate buffer saline (PBS; 0.01 M) and blocked using blocking solution (10% normal donkey serum, 1% bovine serum albumin (BSA; w/v), 0.5% triton in PBS) for 60 min. The sections were incubated overnight with primary antibodies (Table 1) diluted in blocking solution with 0.01% sodium azide overnight Sections were washed with PBS and incubated with secondary antibodies at 1:500 dilution for 1 h. Secondary antibody concentrations, sections used and technical details are indicated in Table 3. Nuclear staining was performed by incubating the sections with Hoechst 33258, (1:5,000 dilution; Thermo Fisher Scientific; Cat#: H3569; RRID:AB_2651133) for 5 min, before three 5 min washes in distilled water. Sections were mounted using ProLong Gold Antifade Mountant (Thermo Fisher Scientific; Cat#: P36934; RRID:AB_015961).

**TABLE 3:** Secondary antibodies used for immunostaining.

Figure	Primary antibody	Secondary antibody	RRID	Tissue	Microscope
Figure 6; Cx/Hc/Cb	BMP2	Cy3 donkey-anti-rabbit	AB_2307443	Fresh frozen 40 μm	LSM 510
	BMP4	Cy3 donkey-anti-rabbit	AB_2307443		
	GFAP	Cy2 donkey-anti-chicken	AB_2340370		
Figure 6—Mb	BMP2	Cy3 donkey-anti-rabbit	AB_2307443	Fresh frozen 20 μm	AxioScan Z1
	BMP4	Cy3 donkey-anti-rabbit	AB_2307443		
	GFAP	Cy5 donkey anti-chicken	AB_2340674		
	DAT	Cy2 donkey anti-rat	AB_2340674		
Figure 7	BMP2	Cy2 donkey anti-rabbit	AB_2340612	Fresh frozen 20 μm	LSM 710
	BMP4	Cy2 donkey anti-rabbit	AB_2340612		
	DyLight 594 Tomato Lectin	Vector Laboratories	AB_2336416		
Figure 8	BMP4	Cy3 donkey-anti-rabbit	B_2307443	Fresh frozen 40 μm	LSM 710
	GFAP	Cy5 donkey anti-chicken	AB_2340674		
Figure 9 and Supplementary Figure 2	AQP4	Cy3 donkey-anti-rabbit	AB_2307443	Perfused 14 μm	LSM 710
	GFAP	C2 donkey-anti-chicken	AB_2340370		
	pSmad1/5/9	Cy3 donkey-anti-rabbit	AB_2307443		
Supplementary Figure 1	BMP2	Cy2 donkey anti-rabbit	AB_2340612	Fresh frozen 40 μm	LSM 510
	BMP4	Cy2 donkey anti-rabbit	AB_2340612		
	NeuN	Cy3 donkey anti-mouse	AB_2315777		
	MAP2	Cy3 donkey anti-chicken	AB_2340363		

**FIGURE 1:**
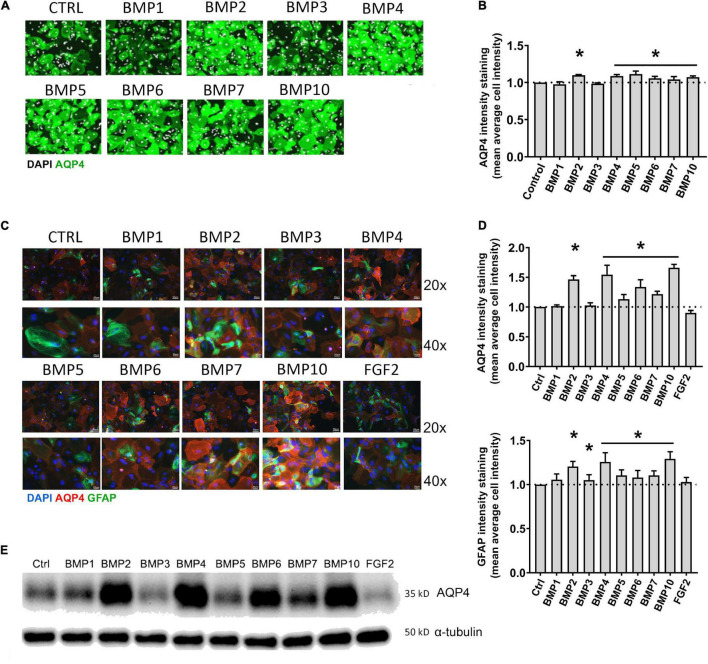
BMPs upregulate AQP4 in mESC astrocytes and primary mouse astrocytes after 6 days treatment. **(A)** Plate Runner assay on mESC derived astrocytes treated with BMPs and immunostained with an antibody to AQP4 demonstrate stronger immunofluorescence intensities (green) in calls treated with BMPs –2, –4, –5, –6, –7, and –10. AQP4 staining shown in green, nuclear staining with DAPI is shown in white pseudocolor. **(B)** Assessment of fluorescence intensity reveals a significant increase in AQP4 staining in cells treated with BMPs –2, –4, 5, 6, 7, and 10 when comparing mean average cell intensities. The AQP4 immunofluorescent intensity in cells treated with BMP1 and BMP3 is not different from that of the untreated cells (CTRL). Staining intensity is presented as fold changes of mean average cell intensity normalized to that of untreated cells. *Sig ANOVA LSD *post-hoc* test, horizontal lines with * indicates significance in several samples; error bars: SEM; *P* < 0.05; *n* = 4. **(C)** Plate Runner assay on matured primary astrocytes treated with BMPs and immunostained with antibodies to AQP4 (red) and GFAP (green) demonstrates stronger immunofluorescence intensities in cells treated with BMPs –2, –4, –5, –6, –7, and –10. AQP4 staining shown in red, nuclear staining with DAPI shown in blue, GFAP staining shown in green with 20× and 40× magnification. **(D)** Assessment of fluorescence intensity in treated primary astrocytes reveals a significant increase in AQP4 and GFAP staining in cells treated with BMPs –2, –4, –5, –6, –7, and –10 when comparing mean average cell intensities. BMP1, BMP3, and FGF2 do not affect AQP4 staining intensities Staining intensity is presented as fold changes of mean average cell intensity normalized to that of untreated cells (CTRL). *Sig ANOVA LSD *post-hoc* test, horizontal lines with *indicates significance in several samples; error bars: SEM; *P* < 0.05; *n* = 4. **(E)** Representative immunoblot of primary astrocytes treated with BMPs demonstrates that AQP4 expression on the protein level is increased by BMPs –2, –4, –6, –7, and –10, while corresponding α-tubulin expression is not affected. Samples from two separate experiments were run in parallel in the same gel with identical results, *n* = 2.

**FIGURE 2:**
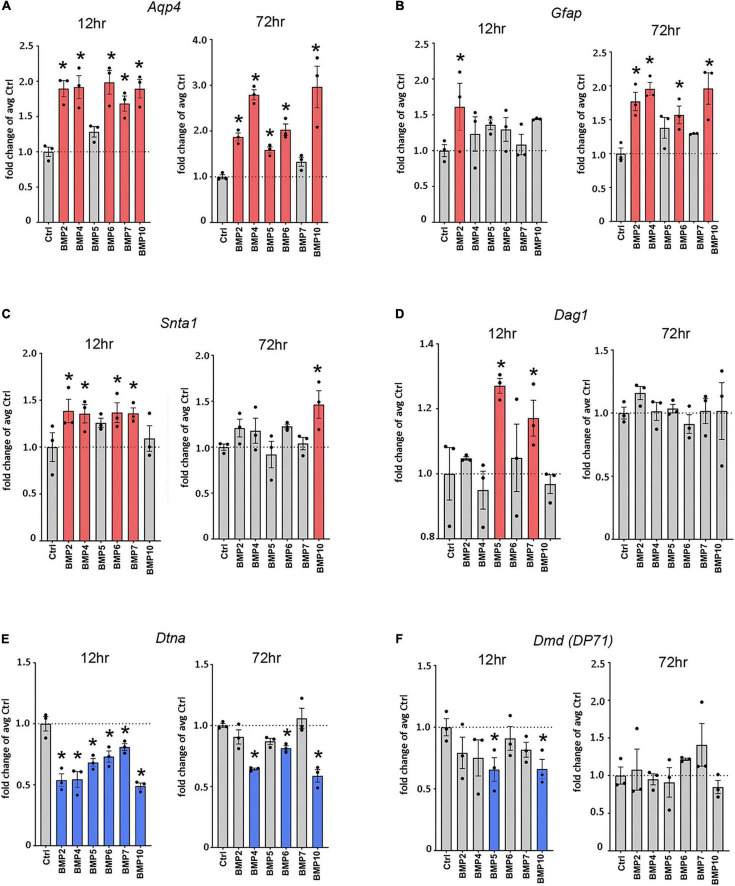
Canonical BMPs transcriptionally regulate *Aqp4*, *Gfap*, and DAP complex genes. RT-qPCR of *Aqp4* **(A)**, *Gfap* **(B)**, DAP complex genes *Snta1* **(C)**, *Dag1* **(D)**, *Dtna* **(E)**, and *Dmd* **(F)**; (DP71 transcript variant) on cDNA from BMP treated primary mature astrocytes confirms transcriptional regulation by canonical BMPs. Transcripts differentially regulated by BMPs at 12 and 72 h. Expression is shown as fold changes compared to untreated cells (Ctrl). Red coloring indicates significant upregulation; Blue coloring indicates significant downregulation. *Sig ANOVA LSD *post-hoc* test; error bars: SEM; dots: individual sample values; *P* < 0.05; *n* = 3.

**FIGURE 3:**
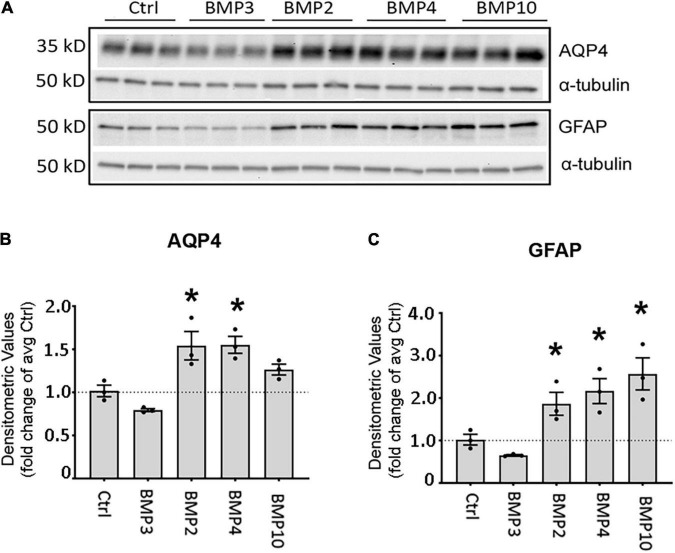
Canonical BMPs increase expression of AQP4 and GFAP protein in astrocytes. **(A)** Immunoblot assessment of AQP4 and GFAP protein lysates after treatment with BMPs for 72 h validates upregulation on the protein level only in cells treated with BMPs known to activate Smad1/5/9 (BMPs –2, –4, –10) and not the non-canonical BMP3. **(B,C)** Densitometric analysis of the immunoblots confirms that both AQP4 and GFAP are significantly upregulated by BMP2, BMP4 and BMP10 treatment. Expression shown as fold changes compared to untreated cells (Ctrl). *Sig ANOVA LSD *post-hoc* test; error bars: SEM; dots: individual sample values; *P* < 0.05; *n* = 3.

**FIGURE 4:**
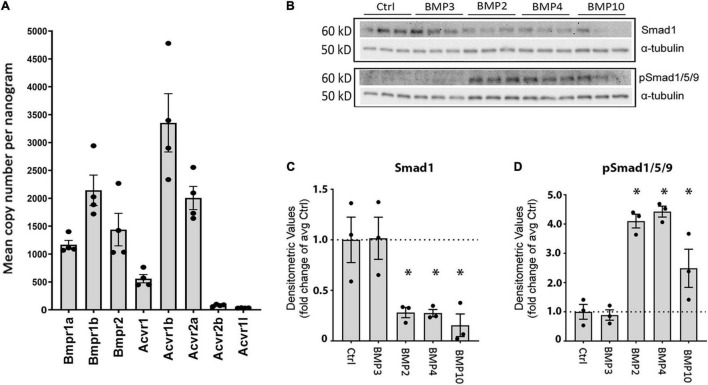
Matured primary astrocytes express BMP receptors and reveal activation of Smad1/5/9 upon treatment with canonical BMPs. **(A)** RT-qPCR confirms that astrocytes express BMP receptor transcripts. BMP receptor transcripts *Bmpr1a*, *Bmpr1b*, *Bmpr2*, *Acvr1*, *Acvr1b*, and *Acvr2a* are robustly expressed, while *Acvr1* exhibits low expression and *Acvr1l* is not expressed. Values are presented as mean copy numbers per nanogram total RNA input into the cDNA reaction. Dots: individual sample values; Error bars = SEM; *n* = 4. **(B–D)** Immunoblots and quantification using antibodies to Smad1 and phosphorylated Smad1/5/9 (pSmad1/5/9) on protein lysates from primary astrocytes after 3 days of BMP treatment. α -tubulin was used as a loading control. *Sig ANOVA LSD *post-hoc* test; Dots: individual sample values; Error bars = SEM; *P* < 0.05; *n* = 3. **(C)** Smad1 is expressed in all samples and significantly lower in cells treated with BMP2, –4, and –10. **(D)** pSmad1/5/9 phosphorylation is induced and significantly increased only in samples treated with canonical BMPs –2, –4, and –10.

**FIGURE 5:**
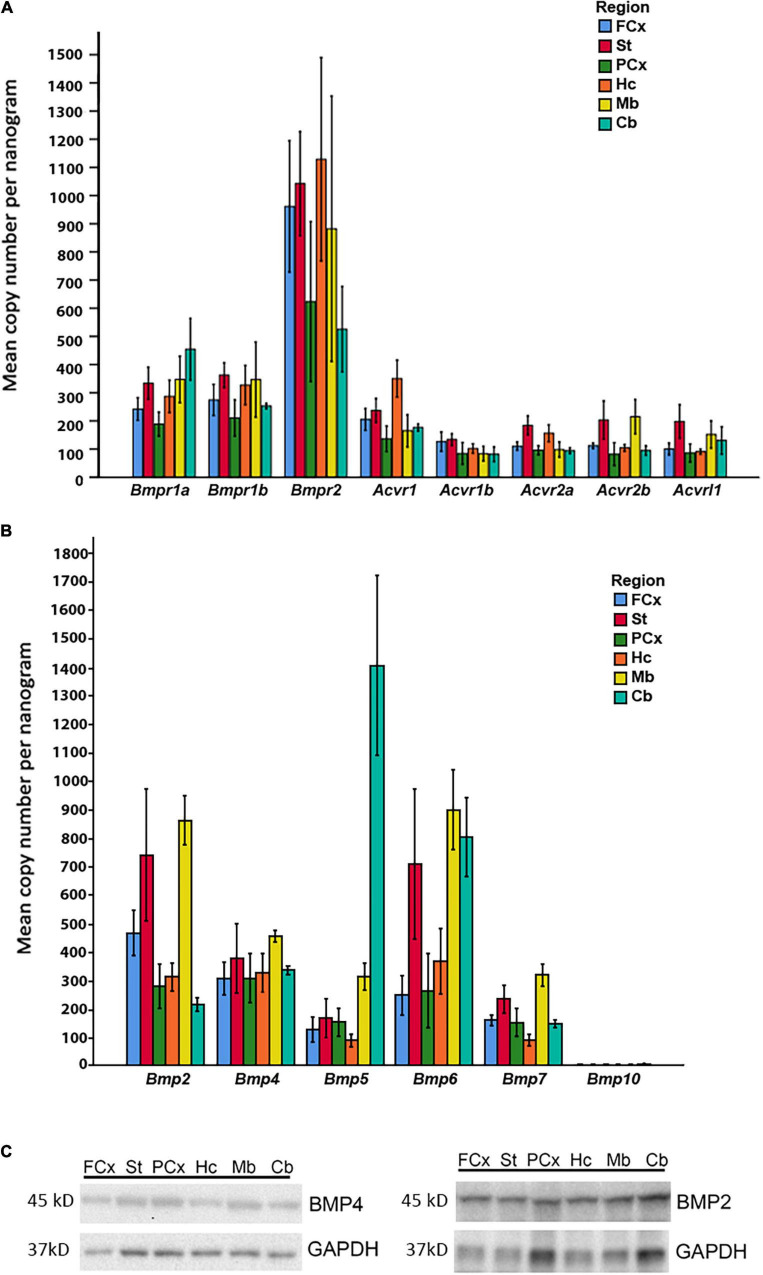
Expression of BMP receptors and canonical BMPs in different brain regions. **(A)** RT-qPCR demonstrates expression of all Bmp receptor transcripts in different regions of adult mouse brain. *Bmpr1a* and *Bmpr1b* transcripts are moderately expressed, while *Bmpr2* transcripts exhibit particularly high expression in all regions. *Acvr1*, *Acvr1b*, *Acvr2a*, *Acvr2b*, and *Acvrl1* transcripts all show low to moderate expression. Values presented as copy numbers per nanogram total RNA input into the cDNA reaction, Error bars = SEM; *n* = 4. **(B)** RT-qPCR demonstrates that all canonical BMPs, with the exception of *Bmp10*, are expressed in all brain regions investigated. *Bmp2* transcripts are expressed throughout the brain, with highest levels in striatum and midbrain. *Bmp4* transcripts are moderately expressed throughout all regions. *Bmp5* transcripts are moderately expressed throughout the brain, but highly expressed in cerebellum. *Bmp6* transcripts are highly expressed in striatum, midbrain and cerebellum, while expression is moderate in other regions. *Bmp7* transcripts are moderately expressed in all regions. *Bmp10* transcripts were not detected in any of the investigated regions. Values presented as copy numbers per nanogram total RNA input into the cDNA reaction- Error bars = SEM; *n* = 4. **(C)** Representative immunoblot shows expression of BMP2 and BMP4 protein in the investigated brain regions in samples from the same animals, *n* = 4.

**FIGURE 6:**
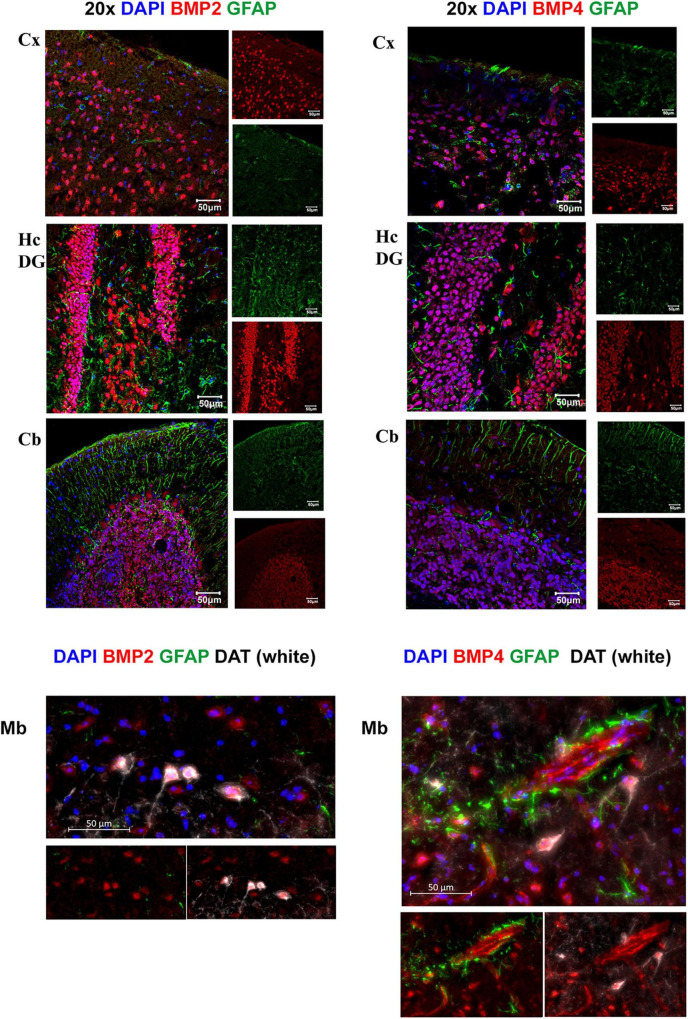
BMP2 and BMP4 are mainly localized in neurons. Confocal images showing BMP2 and BMP4 (in red), the astrocyte marker Glial fibrillary acidic protein (GFAP; in green). Nuclear staining is shown in blue. BMP2 and BMP4 is expressed in neurons in cortex, hippocampus, cerebellum and midbrain. The staining does not co-localize with GFAP in the investigated regions. In midbrain, BMP2 and BMP4 expression is seen in dopaminergic neurons (DAT; white), while BMP4 staining is also observed in vessels. Cx, neocortex; Hc CA, hippocampus CA1 region; Hc DG, hippocampus dentate gyrus; Cb, cerebellum; Mb, midbrain. Scale bars = 50 μm.

**FIGURE 7:**
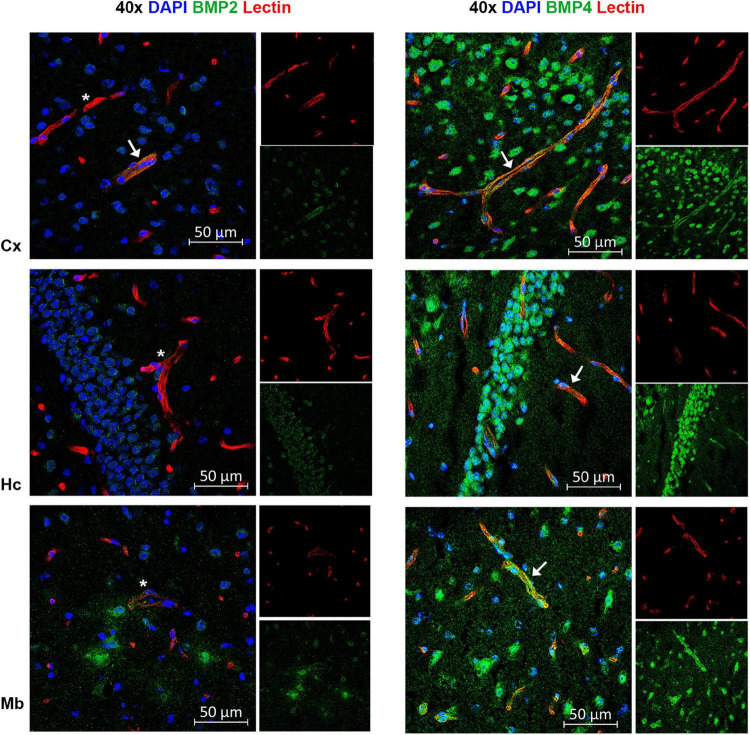
A subpopulation of endothelial cells express BMP2 and BMP4. Confocal images showing BMP2 and BMP4 (green) and the endothelial cell marker tomato lectin (red). Nuclear staining is shown in blue. In some vessels in neocortex, BMP2 staining is observed (arrow), while most vessels are BMP2 negative (asterisks) in neocortex, hippocampus and midbrain. In contrast, widespread BMP4 staining is observed in vessels in cortex, hippocampus and midbrain (arrow). Cx, neocortex; Hc CA, hippocampus CA1 region; Mb, midbrain. Scale bars = 50 μm.

**FIGURE 8:**
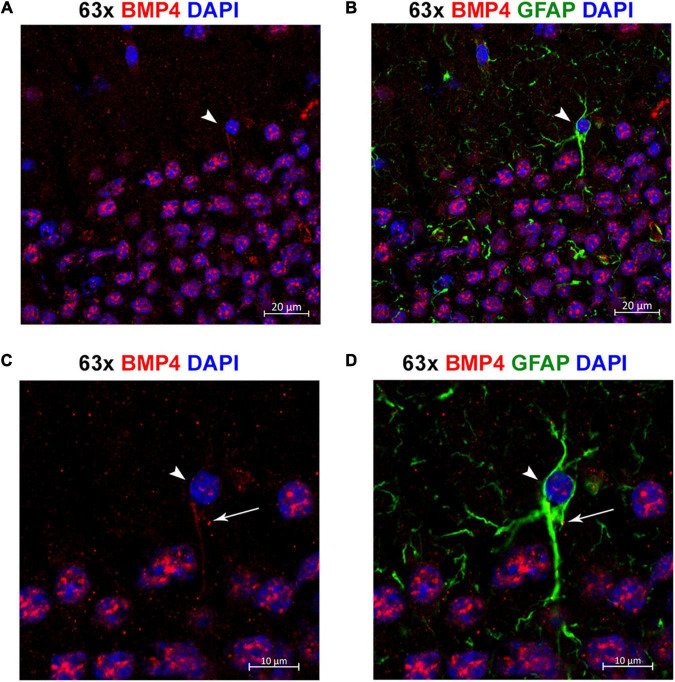
Individual astrocytes express BMP4. **(A,B)** Confocal images identifying BMP4 staining in hippocampal astrocytes. Strong BMP4 labeling (red) is localized around the nuclei (blue) of hippocampal neurons, with diffuse and punctate labeling in the parenchyma and weaker labeling in a GFAP (green) positive astrocyte cell body (arrowhead). **(C,D)** Magnified images focused on the individual astrocyte from **(A,B)** exhibiting BMP4 labeling mainly localized to the area around the nucleus (arrowhead) with some labeling also present in processes (arrow). Scale bars = 20 μm **(A,B)**, 10 μm **(C,D)**.

**FIGURE 9:**
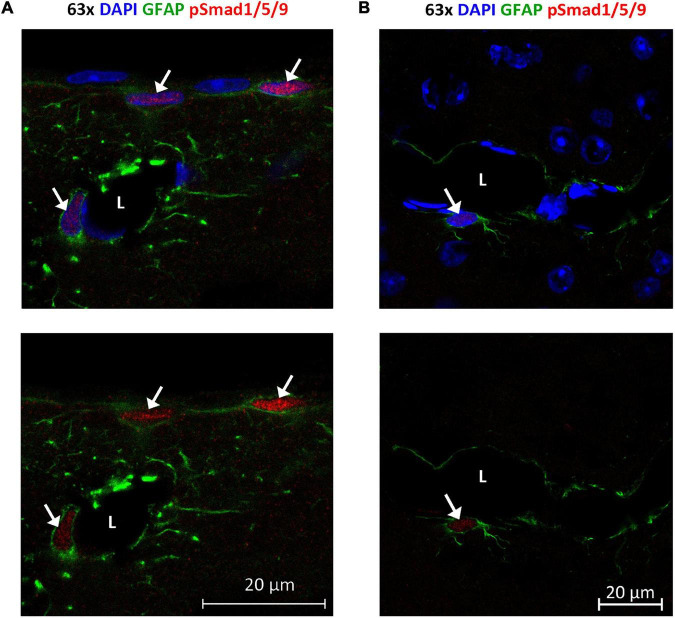
Phosphorylated Smad1/5/9 is mainly localized in subpial and perivascular astrocytes. **(A,B)** Confocal images, Upper panel with DAPI nuclear staining confirm immunofluorescent phospho-Smad1/5/9 staining in astrocytes. **(A)** Astrocytes on the cortical surface and in association with blood vessels are positive (arrows) for nuclear phospho-Smad1/5/9 (red), while endothelial cells and pial cells are not. **(B)** Phospho-Smad1/5/9 (red) staining is present in the nucleus of a GFAP-positive astrocyte associated with a vessels (arrow), but nuclei belonging to endothelial cell lining the vessel, or nuclei in other GFAP-negative cells (some of them with a clear nucleolus) exhibit only very faint or no staining at all. L, vessel lumen. Scale bars = 20 μm.

### Imaging

Images were acquired using a LSM 510 META Confocal Microscope (Zeiss, Jena, Germany) and a LSM 710 confocal microscope (Zeiss, Jena, Germany) at 20× (air objective), 40× (oil objectives) or 63× (water objective) magnification. AxioScan Z1 (Carl Zeiss) was used to acquire scanned immunofluorescence images from midbrain. The ZEN 2.6 (blue edition) (Carl Zeiss Microscopy GmbH, 2018) software was used for image processing.

## Results

### Bone Morphogenetic Proteins (2, 4, 5, 6, 7, and 10) Increase Aquaporin-4 in Mouse Embryonic Stem Cells-Derived and Primary Mouse Astrocytes

We treated mouse embryonic stem cell (mESC)-derived astrocytes with 50 ng/μL BMPs for 6 days, immunostained with antibody to AQP4 and performed a Plate Runner assay (Figure 1A). Assessment of fluorescence intensity revealed a significant increase in AQP4 staining in cells treated with BMP2 (1.36-fold, *p* < 0.0001), BMP4 (1.36-fold, *p* < 0.0001), BMP5 (1.33-fold, *p* < 0.0001), BMP6 (1.35-fold, *p* < 0.0001), BMP7 (1.34-fold, *p* < 0.0001), BMP10 (1.37-fold, *p* < 0.0001), whereas no change was found in cells treated with the non-canonical BMP1 and BMP3, compared to untreated controls (Figure 1B).

To investigate whether matured primary astrocytes in culture show similar responses to BMP treatment, primary mouse astrocytes were matured 2 weeks after the first split (P1) and treated with the same panel of BMPs. FGF2 was included as a negative control (Savchenko et al., 2019). Cells were then fixed and immunostained with antibodies to AQP4 and Glial fibrillary acidic protein (GFAP), the latter allowing to specifically identify astrocytes (Figure 1C). Immunofluorescence signal for AQP4 was increased in cells treated with the canonical BMPs (BMP 2, 4, 5, 6, 7, and 10; Figure 1D). The strongest increases were found after treatment with BMP2, BMP4 and BMP10. Notably, BMP2 (1.46-fold, *p* < 0.0001), BMP4 (1.54-fold, *p* < 0.0001) and BMP10 (1.66-fold, *p* < 0.0001) increased AQP4 expression by ∼50% compared to untreated control cultures. GFAP expression was also affected by these BMPs (Figure 1D), showing a 20–30% increase after BMP2 (1.21-fold, *p* < 0.0001), BMP4 (1.26-fold, *p* < 0.0001) and BMP10 (1.29-fold, *p* < 0.0001) treatments, compared to controls. BMP3 did not significantly affect AQP4 expression (*p* = 0.658), but led to a 1.05-fold increase in GFAP (*p* = 0.029). FGF2 did not significantly affect AQP4 (FGF2 *p* = 0,083) or GFAP expression (*p* = 0.206).

Western blots of astrocyte lysates confirmed the results of the immunofluorescence analysis, showing that BMP2, BMP4, and BMP10 were the most effective in causing an increase in AQP4 protein levels, followed by BMP6 (Figure 1E). As with the mESC cells, BMPs 1 and 3 did not alter AQP4 expression in primary astrocytes indicating a conserved mechanism in embryonic and matured primary astrocytes.

### Bone Morphogenetic Proteins Differentially Regulate Transcription of *Aqp4*, *Gfap*, and Genes Encoding Members of the Dystrophin-Associated Protein Complex

To assess whether the effects of BMPs on AQP4 and GFAP occur at the transcriptional level, we performed RT-qPCR on mRNA from primary astrocyte cultures treated with BMPs for 12 and 72 h (Figure 2) and untreated controls. Our data showed that *Aqp4* was rapidly and consistently upregulated by BMP2 (12 h 1.89-fold, *p* < 0.001; 72 h 1.87-fold, *p* = 0.002), BMP4 (12 h 1.92-fold *p* < 0.001; 72 h, 2.79-fold, *p* < 0.001), BMP6 (12 h 1.98-fold *p* < 0.001; 72 h 2.0-fold *p* < 0.001) and BMP10 (12 h 1.89-fold *p* < 0.001; 72 h 2.96-fold *p* < 0.001), compared to untreated control cells. BMP5 (1.59-fold *p* = 0.026) and BMP7 (1.68-fold *p* < 0.001) induced upregulation at 72 and 12 h, respectively. The strongest inductive effect after 72 h was observed with BMP10, which causes a threefold increase in *Aqp4* expression (Figure 2A). Our data further showed that at 12 h after treatment, *Gfap* is strongly upregulated by BMP2 (1.61-fold, *p* = 0.014), and by 72 h, BMP2 (1.78-fold *p* = 0.001), -BMP4 (1.96-fold *p* < 0.001), BMP6 (1.57-fold *p* = 0.01) and BMP10 (1.96-fold *p* < 0.001) upregulated *Gfap* transcript levels compared to untreated cells (Figure 2B).

Next, we investigated the effect of BMPs on expression of the DAP complex genes products which are required for anchoring of AQP4 to astrocytic endfoot membrane domains. We found that the expression of α-syntrophin (*Snta1*) was upregulated by BMP2 (1.39-fold *p* = 0.013), BMP4 (1.36-fold, *p* = 0.02), BMP6 (1.37-fold *p* = 0.017) and BMP7 (1.36-fold *p* = 0.019), compared to untreated cells at 12 h and by BMP10 (1.46-fold *p* = 0.007) at 72 h compared to untreated cells (Figure 2C). We also found significant upregulation of *Dag1* by BMP5 (1.27-fold *p* = 0.002) and BMP7 (1.17-fold *p* = 0.037) at 12 h compared to untreated cells, with no detectable effects at 72 h (Figure 2D). The *Dag1* transcript gives rise to two protein products, α- and β-dystroglycan which might be differentially regulated and cannot be separately assayed by RT-qPCR (Holt et al., 2000).

Interestingly, expression level of both α-dystrobrevin (*Dtna*) and dystrophin isoform DP71 (*Dmd*) were downregulated by BMPs (Figures 2E,F). In the case of *Dtna*, all BMPs tested caused a downregulation at 12 h (BMP2 0.54-fold, BMP4 0.54-fold, BMP5 0.68-fold, BMP6 0.73-fold *p* = 0.001, BMP7 0.86-fold *p* = 0.008, and BMP10 0.49-fold, *p* < 0.001) compared to untreated controls, while the effects of BMP4 (0.64-fold *p* < 0.001), BMP6 (0.81-fold *p* = 0.016) and BMP10 (0.59-fold *p* < 0.001) persist at 72 h (Figure 2E). The *Dmd* transcript variant DP71 was significantly downregulated by BMP5 (0.66-fold *p* = 0.022) and BMP10 (0.66-fold *p* = 0.024) at 12 h compared to controls, whereas no significant effects were detected at 72 h (Figure 2F).

These results indicate that BMPs can regulate astrocytic gene expression, and that this regulation has different temporal profiles.

### Bone Morphogenetic Proteins Treatment of Primary Astrocytes Also Upregulates Aquaporin-4 and Glial Fibrillary Acidic Protein at the Protein Level

As BMP2, BMP4, and BMP10 consistently showed strong induction of AQP4 and GFAP transcripts at both time points, we chose to validate these effects at the protein level after 72 h treatment (Figure 3A). We found that AQP4 protein expression was significantly increased after treatment with BMPs that signal through the Smad1/5/9 signaling pathway. Both BMP2 (1.54-fold, *p* = 0.003) and BMP4 (1.55-fold, *p* = 0.003) increased AQP4 levels compared to controls (Figure 3B). In the case of GFAP, protein levels were doubled after treatment with BMP2 (1.86-fold *p* = 0.041), BMP4 (2.16-fold *p* = 0.01) and BMP10 (2.57-fold *p* = 0.002) compared to controls (Figure 3C). No such effect was induced by BMP3 (Smad2/3 signaling pathway) on AQP4 (*p* = 0.130) or GFAP (*p* = 0.328), which we used as a negative control (Figures 3B,C).

### Primary Astrocytes Express Bone Morphogenetic Proteins Receptors and Bone Morphogenetic Proteins Treatment Activates the Smad1/5/9 Pathway

We next investigated whether astrocytes expressed BMP receptors and whether the associated Smad1/5/9 pathway was activated in them. Using RT-qPCR, we found that primary astrocytes in culture robustly express *Bmpr1a, Bmpr1b, Bmpr2, Acvr1, Acvr1b, and Acvr2a* BMP receptors (Figure 4A). Interestingly, expression of *Acvr2b* was low, and that of *Acvr1l* was absent.

Furthermore, we found that Smad1 is present in astrocytes and that Smad1/5/9 is phosphorylated upon treatment with BMPs –2, –4, and 10 but not by BMP3 which is known to signal through the Smad2/3 pathway (Figure 4B). Quantification shows that Smad1 expression is significantly lower (Figure 4C) and Smad1/5/9 phosphorylation is significantly higher (Figure 4D) in cells treated with BMP2, –4, –10 compared to the untreated or BMP3 treated cells. This is consistent with the idea that both AQP4 and GFAP expression is regulated by the Smad1/5/9 signaling pathway in astrocytes.

### *Bmp* Transcripts and Receptors Are Expressed in Adult Mouse Brain With Regional Heterogeneity

In light of our *in vitro* data, we further examined whether the relevant BMP receptors were expressed in adult mouse brain. We found that all BMP receptors were expressed (Figure 5A). *Bmpr1a* and *Bmpr1b* showed moderate to high expression. Notably, *Bmpr2* expression was high in all regions, while *Acvr1*, *Acvr1b*, *Acvr2a*, *Acvr2b*, and *Acvrl1* were weakly to moderately expressed.

We then asked whether the adult mouse cells in brain expresses BMPs known to be associated with the Smad1/5/9 pathway. *Bmp* transcripts were readily detected in the adult mouse brain, however, regional differences in their expression could be identified (Figure 5B). The RT-qPCR analysis showed moderate to strong expression of *Bmp2*, *Bmp4*, *Bmp5*, *Bmp6*, and *Bmp7* in all brain regions. *Bmp10* expression was not detected in any brain region. Notably, *Bmp2* and *Bmp6* expression was particularly high in the striatum and the midbrain regions. *Bmp4* transcripts appeared to be rather evenly distributed across regions. The *Bmp5* expression level was much higher in the cerebellum than in any other region. To verify that the *Bmp* transcripts were translated into protein in the adult brain, we selected BMP2 and BMP4 for analysis by SDS-PAGE and Western blot. We detected the proteins in all brain regions (Figure 5C).

### Bone Morphogenetic Proteins-2 and -4 Are Mainly Expressed by Neurons

We next assessed the microscopic distribution of BMP2 and BMP4 by use of confocal microscopy combined with various cell type markers in cerebral cortex, hippocampus, cerebellum and midbrain regions. Our results show strong intensity staining of these BMPs in neurons. Indeed, BMP co-localized with neuronal nuclei (NeuN), throughout the brain (Supplementary Figure 1A). The cerebellar Purkinje neurons and granule cells showed also strong BMP2 and BMP4 immunofluorescence (Supplementary Figure 1B). In the midbrain, we found colocalization of BMP2 and BMP4 within both DAT-positive (dopaminergic neurons) and DAT-negative neurons (Figure 6).

### Endothelial Cells in Brain Vessels Differentially Express Bone Morphogenetic Proteins

By co-labeling with BMP antibodies and the endothelial cell marker lectin, we found that BMPs are also present in vessels (Figure 7). BMP4 was expressed in endothelial cells in each of the brain structures analyzed, while BMP2 was identified in neocortical vessels but lacking or very weakly expressed in vessels of the hippocampus and the midbrain.

### Individual Astrocytes Express Bone Morphogenetic Protein 4

By co-labeling with the astrocyte marker GFAP, we identified individual GFAP-positive astrocytes with BMP4 labeling in cell bodies and processes (Figure 8). However, the labeling intensity in individual astrocytes was weak compared with the labeling intensity in adjacent neurons. Moreover, the possibility remains that some of the punctate BMP2 and BMP4 labeling in the neuropil can be attributed to GFAP negative astrocyte processes.

### Nuclei of Subpial and Perivascular Astrocytes Contain Phosphorylated Smad1/5/9 Complex

Finally, we examined localization and activation of Smad proteins. Brain sections were immunostained with phospho-Smad1/5/9 antibody recognizing the activated Smad1/5/9 complex. Immunofluorescence signals mainly occurred in the nuclei of GFAP-positive astrocytes near the brain surface and close to large vessels (Figure 9). Astrocytes near the brain surface typically exhibit high expression of AQP4 both in their subpial endfeet and in the processes extending into the subpial neuropil (Supplementary Figure 2). Only weak or no phospho-Smad1/5/9 signal was observed in endothelial cells and the nuclei of GFAP-negative cells (Figure 8).

## Discussion

AQP4 has long been implicated in several pathological conditions (Verkman et al., 2017; Verkhratsky and Nedergaard, 2018). First and foremost, several lines of evidence identify the perivascular pool of AQP4 as an influx route for water in models of brain edema (Vajda et al., 2002; Amiry-Moghaddam et al., 2003a; Papadopoulos et al., 2004). Furthermore, mislocalization and altered expression of AQP4 is a common denominator in a variety of neurological conditions, ranging from stroke to mesial temporal lobe epilepsy and Alzheimer’s disease (Eid et al., 2005; Frydenlund et al., 2006; Yang et al., 2011; Kitchen et al., 2020).

The extensive knowledge that has been amassed on AQP4 distribution and experimentally induced AQP4 mislocalization contrasts sharply with the dearth of knowledge when it comes to signaling pathways regulating the expression of this water channel. Obviously, identification of these pathways could provide an avenue for new therapies targeting AQP4 in brain. We hypothesized that BMP signaling could be critically involved in the regulation of AQP4. BMPs have long been used to promote differentiation of immature astrocytes (Cheng et al., 2007), and it was recently shown that inhibition of BMP signaling *in utero* reduces the level of AQP4, while overexpression of BMP7 leads to an increase in AQP4 immunofluorescence in the postnatal brain, (Morita et al., 2021). Here we provide evidence that activation of a BMP signaling pathway upregulates AQP4 in matured primary astrocytes.

First, we showed that the effect of BMPs on AQP4 upregulation in mESC derived astrocytes was restricted to a subset of BMPs—namely those activating the canonical Smad1/5/9 pathway. Notably, BMP3—known to activate the alternative pathway through Smad2/3—did not alter AQP4 expression. BMP2, 4, and 10 stood out as the most effective BMPs when it comes to AQP4 upregulation, assessed by immunofluorescence and semiquantitative immunoblotting. The same BMPs—2, 4, and 10—increased the copy number of *Aqp4* mRNA in mature astrocytes, indicating that upregulation occurs at the transcriptional level. Obviously, the relevance of the above findings hinges on two important issues: 1, the BMPs shown to be active *in vitro* must also be released by specific cell types in the mature brain; and 2, the astrocytes must be endowed with relevant BMP receptors. As to point 1 above, we could show that the mature brain expresses ample amounts of BMP2 and 4, as assessed by RT-qPCR and immunocytochemistry. BMP10 was not expressed at significant levels and thus is unlikely to play a role if the blood brain barrier is intact. However, BMP10 occurs in blood and could thus enter the brain under pathophysiological conditions (Jiang et al., 2016; Tillet et al., 2018). Our immunocytochemical analysis showed that BMP2 and 4 are predominantly localized to neurons. Previous data suggest that BMP4 occurs in dense core vesicles indicating that BMP4 (and possibly also other BMPs) belong to a releasable pool (Higashi et al., 2018). BMP4 has been localized to astrocytes in some regions of rat brain (Mikawa et al., 2006; Sato et al., 2010). In the present study we found that BMP4 labeling was restricted to a small subset of GFAP positive cells and processes. However, we cannot rule out that some of the punctate BMP labeling in the neuropil is localized to fine astrocytic processes as these processes often stand out as GFAP negative. BMP2 and BMP4 were expressed in neurons throughout the brain, particularly in neocortex, hippocampus, cerebellum and midbrain. As to point 2 above, our data demonstrate that BMP receptors *Bmpr1a*, *Bmpr1b*, and *Bmpr2* are enriched throughout the adult mouse brain. *Acvr1*, *Acvr1b*, *Acvr2a*, *Acvr2b*, and *Acvrl1* are also widely expressed, but at lower levels. As each of these receptors except *Acvrl1* and *Acvr2b* was shown to occur in primary astrocytes it is likely that the high transcript levels in adult mouse brains reflect BMP receptor expression in astrocytes *in situ*. BMP2 and BMP4 are known to bind mainly to BMPR1A, BMPR1B, and BMPR2 which are highly expressed in all brain regions (Miyazono et al., 2010). Taken together, our findings show that BMPs upregulates AQP4 expression in mature astrocytes *in vitro* and that the essential elements of BMP signaling are present in the adult mouse brain.

### Signaling Pathways Involved in Regulation of Astrocytic Aquaporin-4

BMPs can activate both canonical and non-canonical signaling pathways (Moustakas and Heldin, 2005, 2009). In the canonical signaling pathway, phosphorylated Smad1/5/9 forms a complex with Smad4 and migrates to the nucleus to take part in gene regulation by binding to BMP response elements. The BMPs that are effective when it comes to increase in AQP4 expression led to phosphorylation of Smad1/5/9—suggesting that they act through the canonical Smad1/5/9 pathway. Further, we observed that BMP treatment also led to a dysregulation of members of the dystrophin associated protein (DAP) complex that are equipped with a BMP response element (von Bubnoff et al., 2005). Moreover, in the intact brain, phosphorylated Smad1/5/9 signal was observed mainly in the astrocytic nuclei associated with the pial surface or large vessels. These astrocytes exhibit high levels of AQP4 in their endfoot neuropil processes. Our data strongly suggest that BMPs act through the canonical pathway. However, we cannot rule out the involvement of additional pathways, such as the non-canonical pathway activating p38 MAPK, JNK, and ERK (Moustakas and Heldin, 2005). A study in mice has shown that hypoxia-induced activation of p38 MAPK pathway takes place only in neurons and microglia and not in astrocytes (Bu et al., 2007). However, in rat astrocytes, activation of the p38 MAPK pathway has been linked to upregulation of AQP4 under pathological conditions (Nito et al., 2012; Li et al., 2019). According to one study, rat cortical astrocytes in culture show concomitant upregulation of AQP4 and phosphorylated p38 (pp38) after 16 h of oxygen-glucose deprivation (Nito et al., 2012). In the same study, rats subjected to middle cerebral artery occlusion showed significantly reduced edema formation and infarction size when treated with SB203580, a selective p38 MAPK inhibitor (Nito et al., 2012). While the possible contribution of the p38 MAPK pathway merits further studies it should be emphasized that this pathway may converge with the canonical Smad pathway through its activation of Smad1, as shown in lung cancer cells (Su et al., 2011). Taken together with available literature data our findings suggest that BMPs may play a pivotal role in regulating AQP4 expression in brain.

### Dysregulation of the Dystrophin-Associated Protein Complex in Astrocytes

It has long been known that the DAP complex is critically involved in anchoring of AQP4 to specific astrocyte membrane domains (Frigeri et al., 2001; Neely et al., 2001; Amiry-Moghaddam et al., 2004a). This protein complex interacts with basal lamina components such as laminin and agrin (Hoddevik et al., 2017). AQP4 is lost from endfoot membranes in animals that lack the dystrophin complex (*Dmd*) or the genes encoding the dystrophin associated protein α-syntrophin (*Snta1*) (Frigeri et al., 2001; Neely et al., 2001; Amiry-Moghaddam et al., 2004a,b) or α-dystrobrevin (Lien et al., 2012). As alluded to above, it has previously been shown that the DAP complex members α-syntrophin, DP71 dystrophin and dystroglycan contain BMP response elements (BREs) in their promoter regions, and these are conserved between mouse and human (von Bubnoff et al., 2005). We found that genes encoding for the DAPC members α-dystrobrevin (*Dtna*) and dystrophin (*Dmd*) were downregulated by BMPs, while *Snta1* and *Dag1* were upregulated. This could possibly indicate a “decoupling” of the DAP complex upon activation of the canonical BMP pathway, which would fit with the known loss of astrocyte polarity in neuroinflammation and reactive astrogliosis that is observed in neurodegenerative disorders (Smith et al., 2019). α-syntrophin is intimately coupled to AQP4, presumably through C-terminal PDZ-domains (Neely et al., 2001). The concomitant upregulation of α-syntrophin might provide an important clue to the mechanisms responsible for upregulation of AQP4. In the early postnatal brain, the expression of α-syntrophin precedes the expression of AQP4 and in the mature brain α-syntrophin is the single most important factor determining the size of the AQP4 pool in astrocytic endfeet (Lunde et al., 2015; Hoddevik et al., 2017). In our experiments, *Dtna* was the member of the DAP complex with the most robust downregulation by the canonical BMPs. *Dtna* is a key member of the DAP complex keeping other members of the complex at the perivascular astrocytic endfeet. Mice with genetic deletion of *Dtna* show a complete loss of perivascular AQP4 and a pronounced downregulation of α-syntrophin and dystrophin at this site (Bragg et al., 2010).

Taken together our data suggest that BMP signaling regulates AQP4 expression in astrocytes. BMPs may impact AQP4 along two separate paths: 1, an upregulation of AQP4 expression on the transcriptional level; 2 affecting AQP4 microdistribution through altering the expression levels of DAP complex proteins that under normal circumstances anchor AQP4 to endfoot membranes. Loss of AQP4 polarization combined with a preserved or upregulated AQP4 level is seen in a number of pathophysiological conditions (Eid et al., 2005; Yang et al., 2011; Reeves et al., 2020) and the present study opens for the possibility that this may well be an effect—partly or fully—of perturbed BMP activation. To our knowledge, the present study is the first to demonstrate a mechanism that could be responsible for dysregulation of the DAPC/AQP4 complex and thus explain AQP4 mislocalization in pathological conditions. Our data showed that the same BMPs that caused AQP4 to upregulate also enhanced the expression of GFAP in mature astrocytes. This is in line with previous studies showing an association between BMP4 and astrogliosis (Hart et al., 2020) and a concomitant upregulation of AQP4 and GFAP in several neurodegenerative disorders (Ikeshima-Kataoka, 2016).

## Conclusion

Altered expression or microdistribution of AQP4 is a hallmark of several neurological conditions, prompting for a better understanding of the regulatory pathways involved. Here we show that BMP signaling regulates AQP4 expression, level and localization in mature astrocytes and dysregulates the associated DAP complex by differentially affecting the individual members of this complex. Our data point to the possible involvement of BMPs in conditions where a mislocalization of AQP4 is paired with its preserved or enhanced expression. Alzheimer’s Disease (AD) is a notable example: a decade ago it was shown in a mouse model of AD that AQP4 was conspicuously absent from endfeet adjoining amyloid plaques (Yang et al., 2011), while more recent studies have demonstrated reduced amyloid clearance following targeted disruption of *Aqp4* (Iliff et al., 2012; Xu et al., 2015; Smith et al., 2019; Rosu et al., 2020). AD is associated with upregulation of BMPs and activation of the Smad1/5/9 pathway (Li et al., 2008; Zhang et al., 2021). Further studies should be undertaken to explore whether BMP-induced AQP4 mislocalization contributes to amyloid accumulation by interfering with amyloid clearance through the glymphatic pathway (Rasmussen et al., 2018).

## Data Availability Statement

The original contributions presented in the study are included in the article/Supplementary Material, further inquiries can be directed to the corresponding author/s.

## Ethics Statement

Experimental protocols were approved by the Norwegian Animal Research Authority (NARA) with license number FOTS 8572 and carried out in accordance with the European Directive 2010/63/EU. The derivation of mouse embryonic stem cells (mESC) was approved by the ethical committee for the use of laboratory animals at Lund University and the Swedish Work Environment Authority with permit number M43-15 and carried out in accordance with European and Swedish regulations.

## Author Contributions

MA-M and NS: conceptualization. NS and ES: experimental work and analyses. MA-M and LR: resources. NS, MA-M, and OPO: interpretation, original draft preparation, and writing—review and editing. MA-M: supervision. All authors read and approved the final manuscript.

## Conflict of Interest

The authors declare that the research was conducted in the absence of any commercial or financial relationships that could be construed as a potential conflict of interest.

## Publisher’s Note

All claims expressed in this article are solely those of the authors and do not necessarily represent those of their affiliated organizations, or those of the publisher, the editors and the reviewers. Any product that may be evaluated in this article, or claim that may be made by its manufacturer, is not guaranteed or endorsed by the publisher.
