# Supplementary material for: Canonical Bone Morphogenetic Protein Signaling Regulates Expression of Aquaporin-4 and Its Anchoring Complex in Mouse Astrocytes
Source: Front Cell Neurosci. 2022 Apr 20;16:878154. doi: 10.3389/fncel.2022.878154 (PMC9067306; doi:10.3389/fncel.2022.878154)
Supplement: Supplementary file 1 [file Data_Sheet_1.docx]

Supplementary Material

Figure S1 shows BMP2 and BMP4 localized in NeuN and MAP2 positive neurons.

Figure S2 shows expression pattern of AQP4 in subpial parts of the neocortex


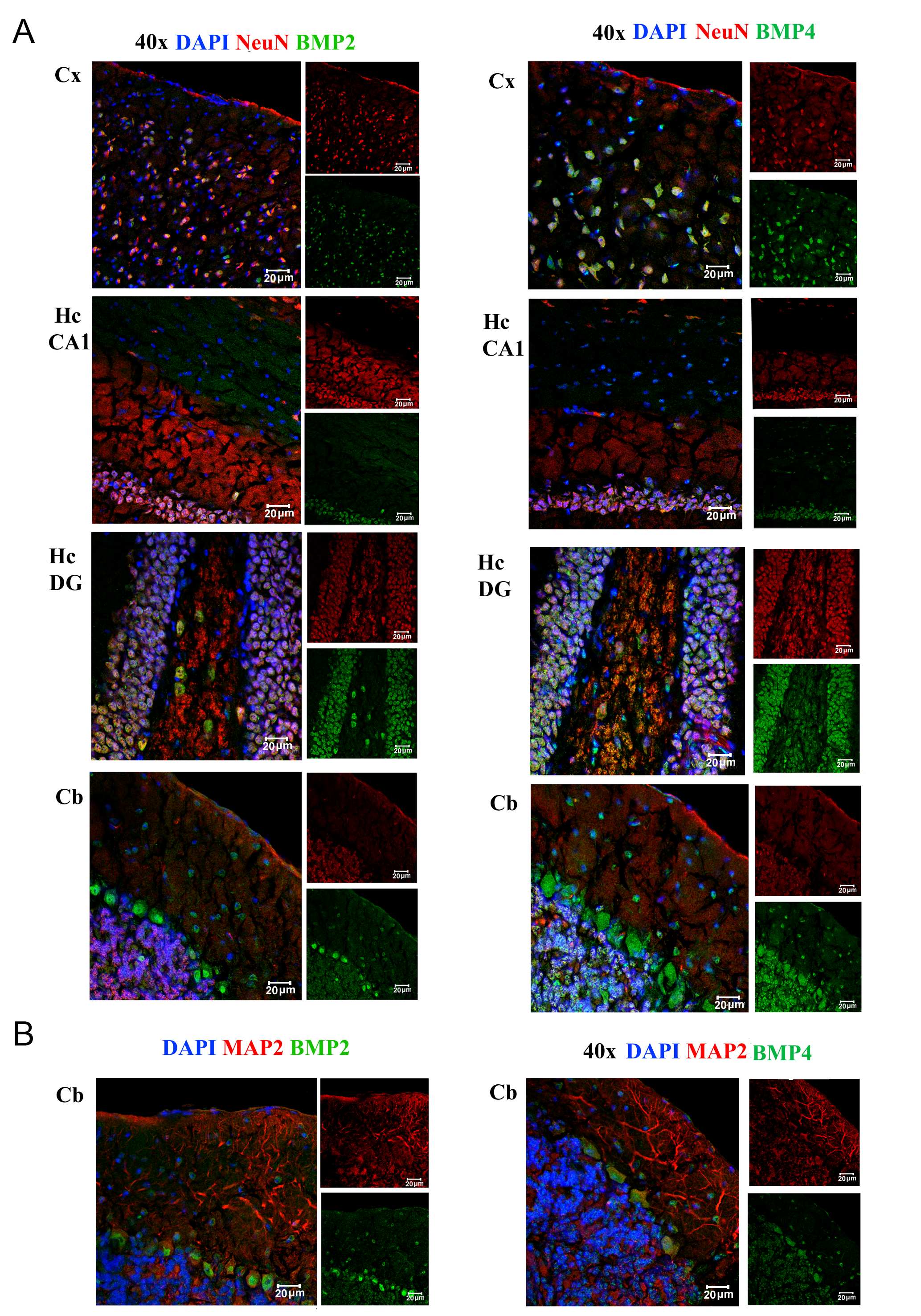


**Supplementary Figure 1 -** **BMP2 and BMP4 are localized in NeuN and MAP2 positive neurons.**

Immunofluorescent confocal microscopy images showing BMP2 and BMP4 (in green), the neuronal nuclei marker NeuN (in red) and microtubule associated protein 2 (MAP2) (in red). Nuclear staining is shown in blue. In neocortex, hippocampus and the cerebellar granular layer, BMP2 and BMP4 co-localize with NeuN. Cerebellar Purkinje neurons are MAP2 positive and show co-localization with BMP2 and BMP4. Cx – neocortex; Hc CA – hippocampus CA1 region; Hc DG – hippocampus dentate gyrus; Cb – cerebellum. Scale bars = 20 μm


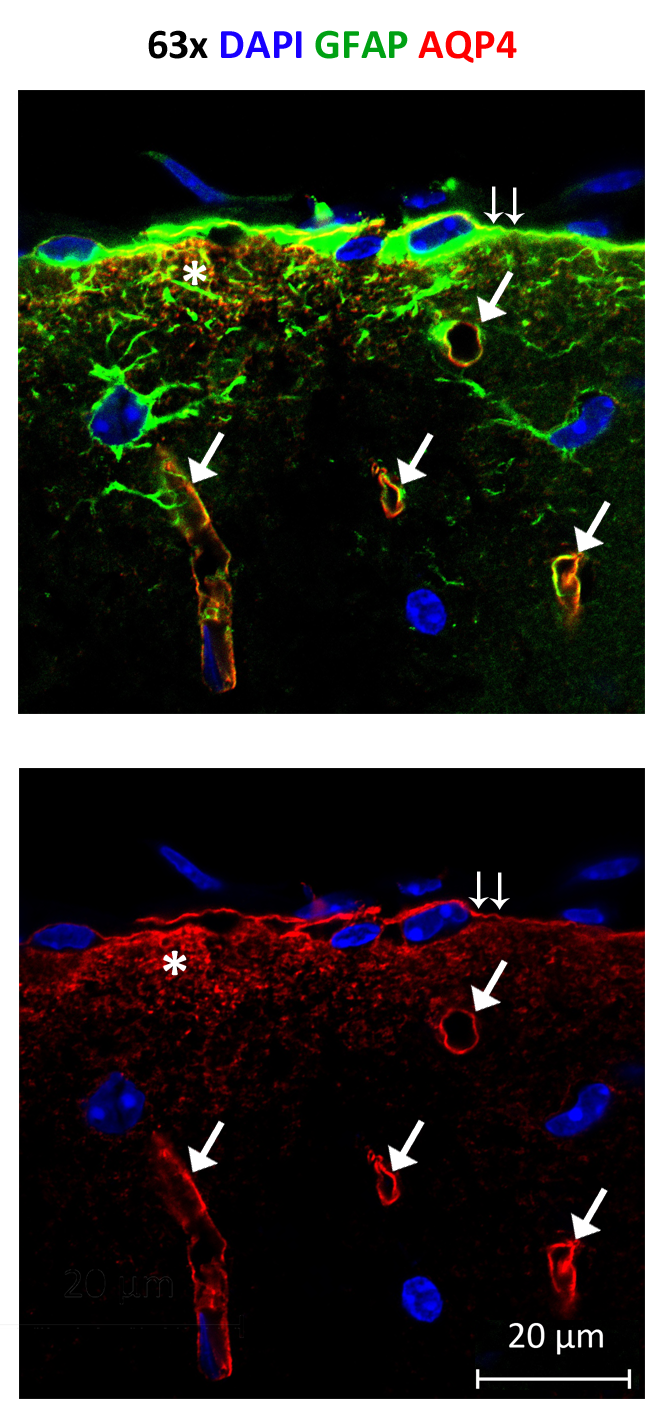


**Supplementary Figure 2- Expression pattern of AQP4 in the subpial parts of the neocortex.**

Confocal immunofluorescent image showing strong AQP4 (red) labeling in the GFAP (green)-positive perivascular (arrows) and subpial endfoot processes (double arrow). The lower panel shows the same image only with AQP4 labeling. AQP4 labeling is also present in the small astrocytic processes extending into the subpial neuropil (asterisk). Nuclear staining is shown in blue*.* Scale bars = 20 μm
